# Visual integration deficits associated with psychosis are independent of diagnosis

**DOI:** 10.1038/s41537-025-00606-0

**Published:** 2025-04-09

**Authors:** Mia Geljic, Matthew Mitchell, Keri-Anne Stevens, Henry Holbrook, Hayley Darke, Patrick Goodbourn, Christina Damicoucas, Mohammad Asghari-Jafarabadi, Suresh Sundram, Olivia Carter

**Affiliations:** 1 Department of Psychiatry, School of Clinical Sciences, Monash University. Monash Medical Centre, 246 Clayton Road, Clayton, VIC 3168 Australia; 2 Melbourne School of Psychological Sciences, Faculty of Medicine, Dentistry and Health Sciences, University of Melbourne, Melbourne, VIC 3010 Australia; 3 Biostatistics Unit, School of Public Health and Preventative Medicine, Faculty of Medicine, Nursing and Health Sciences, Monash University, Melbourne, VIC 3004 Australia; 4 Mental Health Program, Monash Health, Monash Medical Centre, 246 Clayton Road, Clayton, VIC 3168 Australia

**Keywords:** Biomarkers, Psychosis

## Abstract

Evidence of altered visual processing is well‐established in schizophrenia. Visual integration deficits have been highlighted as a potential diagnostic biomarker to distinguish schizophrenia from other psychiatric disorders. Motivated by the current lack of cross-diagnostic assessments of visual integration performance, the current study used the Jittered Orientation Visual Integration (JOVI) task to assess contour integration performance in 85 psychiatric inpatients split into “schizophrenia spectrum” (*n* = 40) and “other psychiatric disorders” (*n* = 45), and healthy controls (*n* = 43). The study also examined attentional and working memory ability using the Digit Span Task. JOVI accuracy scores were found to be significantly impaired relative to healthy controls for both the schizophrenia (*p* < 0.001) and other psychiatric (*p* < 0001) patient groups. In line with a transdiagnostic deficit, no differences in JOVI accuracy were seen between the patient groups (*p* = 0.97) with reduced JOVI accuracy correlating with worsening psychosis regardless of diagnosis (*r* = −0.32, *p* < 0.05). Schizophrenia spectrum patients also showed reduced Digit Span Forward (*p* < 0.001) and Backward scores (*p* < 0.001). The other psychiatric (*p* = 0.024) group were similarly found to be impaired in the Digit Span Backward relative to healthy controls, however no differences were seen between the patient groups. The findings indicate that contour integration deficits are not specific to schizophrenia spectrum disorders, and instead the neurobiological underpinnings of visual integration impairment may share commonality with psychosis more generally. The findings are also consistent with cognitive factors playing a potential role in JOVI performance and highlight the difficulty in teasing apart altered perceptual and cognitive function in psychiatric patient groups.

## Introduction

Schizophrenia, despite its comparatively low prevalence, is a major contributor to global disability^1^. This is because of the chronicity of multiple symptom clusters, notably cognitive impairment^2^.

The majority of individuals with schizophrenia demonstrate moderate-to-severe neurocognitive dysfunction across numerous cognitive and perceptual domains, severely impacting daily functioning^3–6^. A barrier to developing schizophrenia-related neurocognitive dysfunction treatments is the paucity of well-established clinical instruments specifically designed to assess discrete perceptual and cognitive functions characteristically impaired in schizophrenia^7^.

Visual perception impairments are well‐established in schizophrenia^8–11^ and the selective nature of visual processes has identified them as potentially relevant in discriminating schizophrenia from other psychiatric disorders^12^. Low-level visual processing deficits have been highlighted as particularly valuable markers of disease progression as they are believed to be unaffected by higher level cognitive functions and may reflect fundamental differences in neural processing that underpin schizophrenia. Visual integration has been specifically identified as a target to assist in biomarker development in schizophrenia^13^. Visual integration refers to the structuring of low-level visual features (local edges, brightness, motion signals) into increasingly large and complex global percepts^14^ and one paradigm used reliably and routinely to assess this process is contour integration^12^. The task engages top-down processing in the binding of local feature cues into perceptual wholes^15^ and measures participants’ ability to integrate spatially separated elements into a single contour-defined shape^16^.

Previous research into the neural mechanisms involved in contour integration has implicated the primary visual cortex (V1) as integral to perceiving shape contours^17,18^. Prototypical contour integration paradigms sensitively probe visual-integration deficits at the neuronal level via Gabor patches which match the spatial frequency processing properties of orientation-selective V1 neurons^19^.

**Fig. 1:**
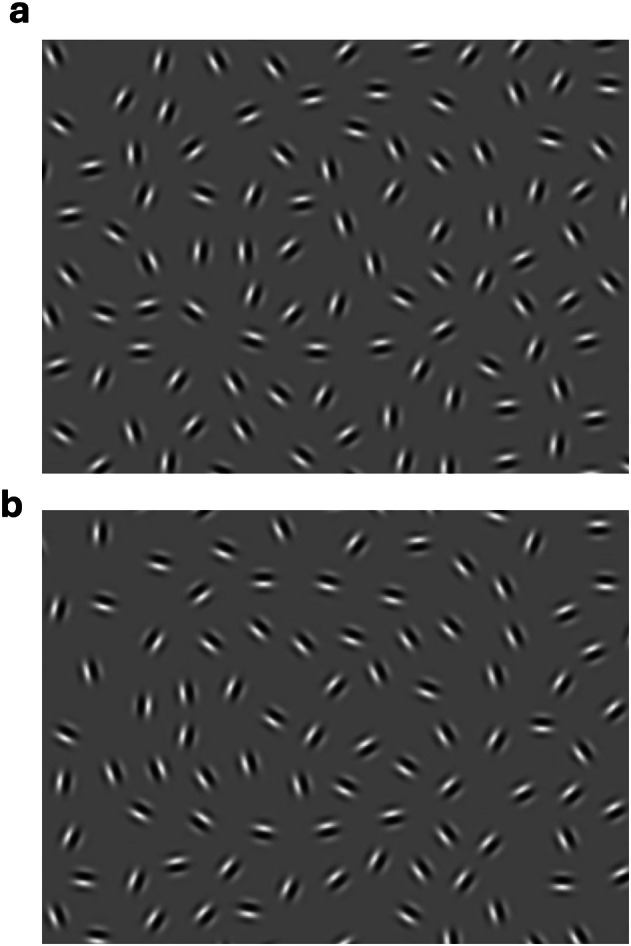
Example JOVI stimuli. **a** Image of a left-ward pointing target stimuli in an easy jitter condition 0° jitter and **b** right-ward pointing target stimulus in the 7°–8° condition.

To assess visual-integration amongst schizophrenia patients in a controlled and reliable way, the Cognitive Neuroscience Test Reliability and Clinical Applications for Schizophrenia (CNTRACS) initiative developed the Jittered Orientation Visual Integration task (JOVI)^20^. The JOVI utilizes “jitter” paradigms (Fig. 1), where successful integration of contour features is dependent on whether the position and orientation of target Gabors varies respectively in a manner consistent (i.e., collinear or co-circular positioning and parallel orientation) or inconsistent (i.e., random positioning and orientation) with the contour path. It is expected that performance for all participants should steadily decrease as a function of increased jitter-magnitude (i.e., task difficulty). However, the magnitude of impairment decrements should be significantly greater in the schizophrenia patient population consistent with a selective deficit in visual-integration hypothesised for this patient group^21^.

Numerous studies have shown evidence of impaired contour integration performance in schizophrenia populations compared to healthy individuals, who are more successful at distinguishing the form of the fragmented shapes^22–29^. However, diagnostic specificity of this impairment has been questioned with similar accuracy-rates being documented between schizophrenia outpatients and schizoaffective and Type 1 bipolar outpatients^30,31^, and no significant between-group JOVI performance differences were documented when schizophrenia inpatients were compared with a mixed psychiatric inpatient population^32^. These data suggest impaired JOVI accuracy may be transdiagnostic, possibly reflecting a shared substrate independent of diagnosis.

In response to these variable findings of JOVI performance across diagnoses, the current study aimed to investigate the JOVI task’s specificity to schizophrenia spectrum disorders by comparing task performance against other psychiatric disorders and symptom profiles. In addition, the study aimed to examine the JOVI task’s validity as a measure of visual-integration ability by addressing potential task confounds, such as attentional and working memory ability.

## Results

### Demographic information

Participant demographic information is presented in Table 1.

**Table 1:** Overall demographic data by diagnostic group.

	Healthy controls	Schizophrenia Spectrum group^a^	Other psychiatric diagnosis group^b^	Statistic for group difference
***n***	43	40	45	-
Age	34.44 (16.41)	37.33 (9.05)	36.89 (9.77)	NS*
Gender (% male)	20 (46.51%)	24 (60%)	21 (46.67%)	NS
Education (years)	12.98 (1.61)	12.3 (2.36)	11.6 (2.17)	*Χ*^2^(2) = 14.27, *p* < 0.01, *n*^2^ = 0.10
Predicted WAIS-IV IQ	114.31 (5.23)	103.14 (10.58)	101.96 (9.89)	*χ*^2^(2) = 39.82, *p* < 0.01, n^2^ = 0.30
PANSS				
Total	-	70.75 (14.05)	60.22 (14.69)	*t*(83) = -3.37, *p* < 0.01, *d* = 0.73
General Psychopathology	-	35.3 (8.21)	32.51 (7.63)	NS
Positive	-	20.18 (5.49)	14.56 (5.39)	*t*(83) = -4.76, *p* < .001, *d* = 1.03
Negative	-	15.38 (5.43)	13.49 (6.25)	NS
Digit Span (*n* = 33)	*n* = 9	*n* = 11	*n* = 13	
Forward	13.56 (3.05)	8.27 (2.28)	10.92 (2.87)	F(2,30) = 9.24, *p* < 0.001, n^2^g = 0.38
Backward	12.44 (1.67)	8.00 (1.73)	9.92 (2.56)	F(2.30) = 11.19, *p* < 0.001, n^2^g = 0.43
Illness Duration (months)	-	138.05 (96.54)	103.30 (89.32)	NS
Chlorpromazine equivalent dose (mg)	-	457.61 (292.06)	325.13 (259.92)	*χ*^2^(2) = 84.58, *p* < 0.01, n^2^ = 0.54
Diazepam equivalent dose (mg)	-	13.38 (10.86)	12.5 (8.48)	NS

Numbers reported are mean and SD, unless otherwise stated.

*NS no statistically significant group differences.

^a^The schizophrenia spectrum disorder group was comprised of first episode psychosis (2), psychotic disorder NOS (1), schizoaffective disorder (13), schizophrenia disorder (24) patients.

^b^The other psychiatric disorder group was comprised of BPAD (18), BPAD with psychotic features (1), BPAD with SIPD (1), BPD (1), brief psychotic disorder with peripartum onset (1), MDD with psychotic features and BPD (1), SIPD (1), SUD and SIPD (1), SUD (1), SIPD and BPD and MDD (1), persistent depressive disorder (1), MDD and PTSD (2), MDD (8), MDD with anxiety (1), MDD and PTSD and BPD (1), BPD and MDD (1), alcohol use disorder (2), and adjustment disorder (2) patients.

Years of education and National Adult Reading Test (NART) estimated Wechsler Adult Intelligence Scale, fourth edition (WAIS-IV) IQ were included as a covariate in subsequent analyses of group differences (see Table 1). Inpatient participants were receiving antipsychotic medication and benzodiazepines during the study, expressed as chlorpromazine equivalents and diazepam equivalents respectively in analyses. Of these, only chlorpromazine equivalents were included as covariates due to significant between-group differences in dosage (χ^2^(2) = 84.58, *p* < 0.01, n^2^ = 0.54, 95% CI [−0.02, 0.04]). No statistically significant differences were found between task performance and medication dose. There were no significant JOVI performance differences between non-psychotic and psychotic non-schizophrenia groups so they were collapsed for further analyses.

### JOVI jitter trials

**Fig. 2:**
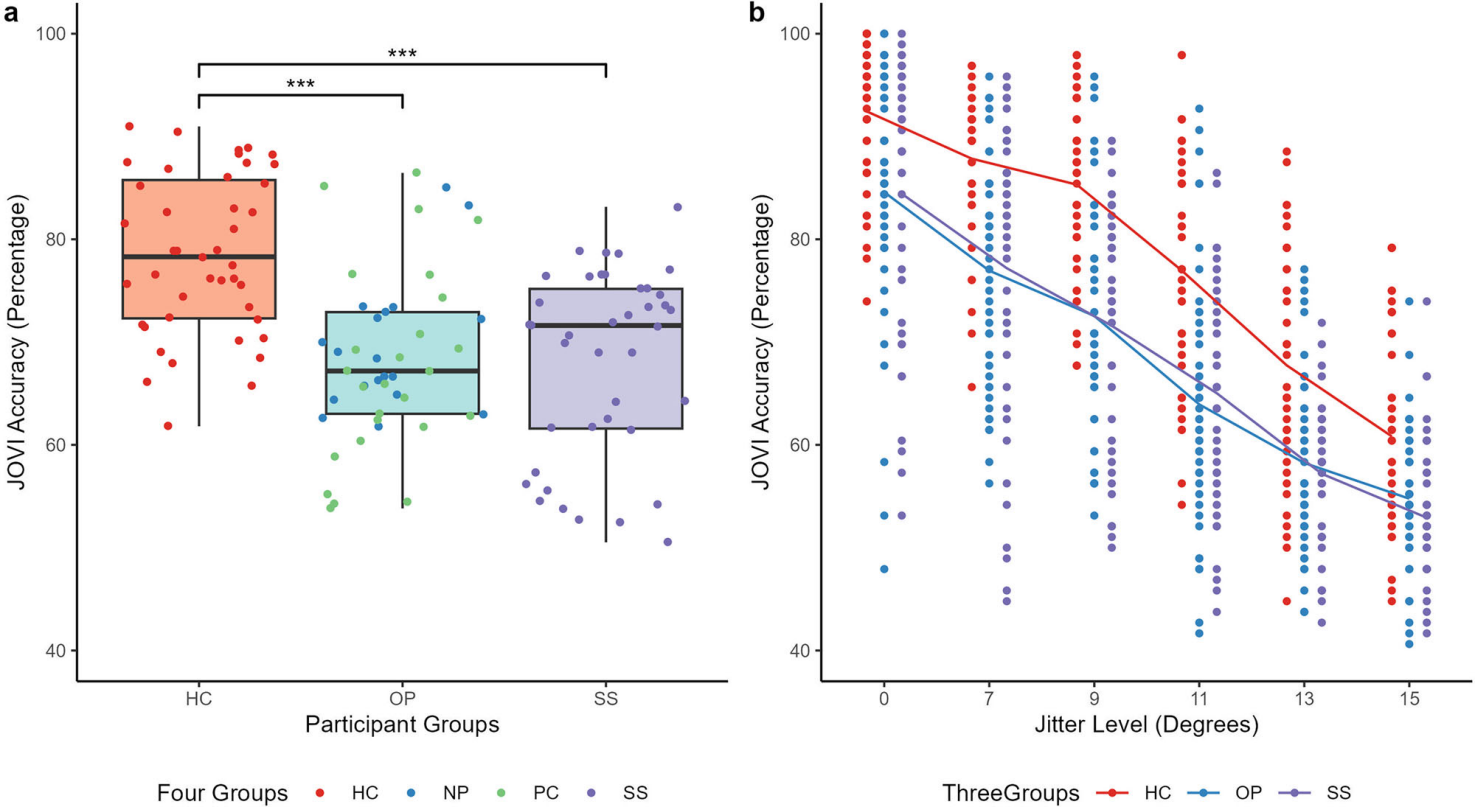
Group comparisons of JOVI performance. **a** Group comparisons of JOVI total percentage accuracy score. Note the dot colouring is for illustrative purposes only, and NP and PC groups were combined into the OP group for statistical analyses. **b** Group comparisons of JOVI percentage accuracy at different jitter levels. HC = healthy controls, OP = other psychiatric inpatients, SS = schizophrenia spectrum inpatients, NP = non-psychosis inpatients, PC = psychosis control inpatients. Boxplot centre line represents median, lower hinge represents 25th percentile, upper hinge represents 75th percentile, lower whisker represents smallest value at most 1.5 × interquartile range (IQR) from the lower hinge, upper whisker represents largest value at most 1.5 × IQR from the upper hinge.

A two-way mixed ANOVA was conducted with accuracy on the JOVI jitter trials (excluding catch trials) as the dependent variable, participant group as the between-subject independent variable and jitter level as the within-subject independent variable. Expectedly, there was a significant main effect of jitter level (F(3.34, 417.57) = 384.43, *p* < 0.001, n^2^g = 0.52, 95% CI [0.72, 1.00]). Tukey’s post-hoc comparisons revealed that jitter trial accuracy declined as the degree of jitter increased. There was a significant main effect of participant group (F(2, 125) = 21.25, *p* < 0.001, n^2^g = 0.18, 95% CI [0.15, 1.00]). Tukey’s post-hoc comparisons revealed that healthy controls had significantly higher accuracy on jitter trials than both the schizophrenia spectrum group (M_D_ = 0.10, *p* < 0.001, n^2^ = 0.21, 95% CI [0.11, 1.00]) and other psychiatric diagnosis group (M_D_ = 0.10, *p* < 0.001, n^2^ = 0.20, 95% CI [0.11, 1.00]). There was no significant difference between the patient groups (M_D_ = 0.00, *p* = 0.97) (Fig. 2a). There was also a significant jitter level and participant group interaction (F(6.68, 417.57) = 2.12, *p* < 0.05, n^2^g = .01, 95% CI [0.00, 1.00]), showing that the effect of jitter trial on accuracy differs between the groups The interaction was such that the schizophrenia spectrum (*p* < 0.0001, n^2^ = 0.17, 95% CI [0.08, 1.00]) and other psychiatric diagnosis (*p* < 0.001, n^2^ = 0.12, 95% CI [0.05, 1.00]) group accuracy was significantly lower at the increased difficulty 9° jitter level than 7° jitter level, whereas healthy controls showed no significant differences in accuracy (*p* = 0.19). Similarly, the schizophrenia spectrum (*p* < 0.05, n^2^ = 0.07, 95% CI [0.01, 1.00]) and healthy control (*p* < 0.0001, n^2^ = 0.17, 95% CI [0.08, 1.00]) group accuracy was significantly lower at the increased difficulty 15° jitter level than 13° jitter level, whereas the other psychiatric group showed no significant difference (*p* = 0.11) (Fig. 2b).

### JOVI threshold analyses

**Fig. 3:**
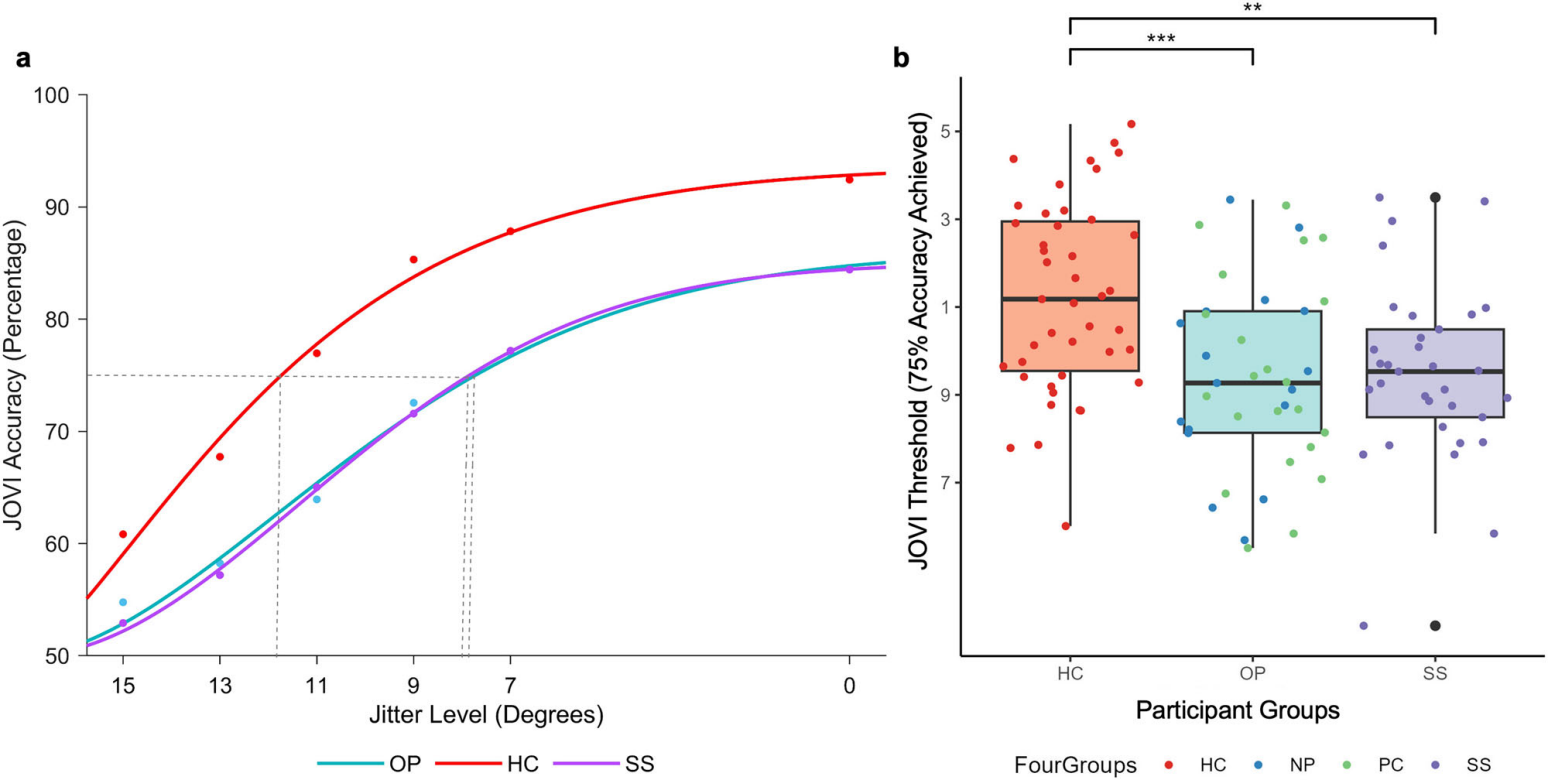
Group comparisons of JOVI mean threshold. **a** Cumulative Weibull distribution function curves fitted to mean group JOVI accuracies at each jitter level. **b** Group comparisons of JOVI Mean Threshold score, i.e. the mean jitter level at which 75% accuracy is achieved. The dot colouring is for illustrative purposes only, and NP and PC groups were combined into the OP group for statistical analyses. HC = healthy controls, OP = other psychiatric inpatients, SS = schizophrenia spectrum inpatients, NP = non-psychosis inpatients, PC = psychosis control inpatients. Boxplot centre line represents median, lower hinge represents 25th percentile, upper hinge represents 75th percentile, lower whisker represents smallest value at most 1.5 × interquartile range (IQR) from the lower hinge, upper whisker represents largest value at most 1.5 × IQR from the upper hinge.

Since standard accuracy-rate can be confounded by inattention and demotivation^30^, findings derived from standard accuracy-based analyses were extended by conducting a curve fitting analysis. Consistent with CNTRICS’ procedure^33^ each participant’s data were fitted with a psychometric curve using the cumulative Weibull distribution function in MATLAB’s curve fitting toolbox. This generates additional information, including jitter slope (how accuracy changes in response to jitter change), jitter threshold (level of jitter orientation at which participants are achieving 75% accuracy) and the upper asymptote parameter (performance on the easiest level of difficulty). First, each participant’s accuracy data were recoded as the amount of distance from a baseline jitter level of 23° at which performance is at chance^33^. This effectively transformed the data to show the conventional monotonically-increasing relationship between the variables. Data from twelve patients could not be fitted to a curve and were removed from subsequent threshold analyses. These twelve patients were comprised of 0 healthy controls and 6 participants of each clinical group, whose poor performance on lower JOVI conditions was unable to fit the curve. Figure 3a shows mean group performance on the JOVI at each jitter level fitted to a Weibull curve in MATLAB. The curves fitted the data as indicated by the high median R-squared values of .94 for all three groups.

Because data was not normally distributed across any of the variables, nonparametric Kruskal-Wallis H tests were used to compare group differences. There was a significant impact of participant group on threshold (χ^2^(2) = 15.82, *p* < 0.001, n^2^ = 0.12, 95% CI [0.03, 0.28]). Bonferroni corrected Wilcoxon rank sum tests revealed that healthy controls had higher threshold parameters compared to the other psychiatric diagnosis group (*p* < 0.01, r = 0.38, 95% CI [0.16, 0.55]) and the schizophrenia spectrum group (*p* < 0.01, r = 0.38, 95% CI [0.18, 0.56]). The median threshold score for healthy controls was 11.18, for the schizophrenia spectrum group was 9.53, and for the other psychiatric group was 9.27 (see Fig. 3b).

There was also a significant impact of participant group on slope (χ^2^(2) = 7.26, *p* < 0.05, n^2^ = 0.05, 95% CI [−0.01, 0.18]). Bonferroni corrected Wilcoxon rank sum tests revealed that healthy controls had a lower slope than other psychiatric diagnosis patients (*p* < 0.05, *r* = 0.28, 95% CI [0.06, 0.48]). The median slope score for healthy controls was 3.75, for the schizophrenia spectrum group was 4.93, and for the other psychiatric group was 5.28. Lower slope scores correspond to steeper slopes and indicate higher sensitivity (i.e. improved accuracy) in response to jitter change as jitter level transitions from hard to easy. Upper asymptote parameters also differed significantly between groups (χ^2^(2) = 10.48, *p* < 0.01, n^2^ = 0.08, 95% CI = [0.01, 0.22]). Bonferroni corrected Wilcoxon rank sum tests showed that the other psychiatric diagnosis group had significantly higher asymptotes compared to the healthy controls (*p* < 0.01, r = 0.35, 95% CI [0.13, 0.55]). The median upper asymptote score for healthy controls was 0.06, for the schizophrenia spectrum group was 0.09, and for the other psychiatric group was 0.14. Higher upper asymptote scores indicate more errors on the easiest jitter condition.

### Positive and negative syndrome scale (PANSS) subscale scores

Table 1 demonstrates independent t-test results for PANSS subscales with Cohen’s d effect sizes. Schizophrenia spectrum patients scored higher on the PANSS Total (M_D_ = 10.53, 95% CI [−16.75, −4.31]) and Positive (M_D_ = 5.62, 95% CI [−7.97, −3.27]) subscales compared to other psychiatric patients.

### Symptom correlations

**Fig. 4:**
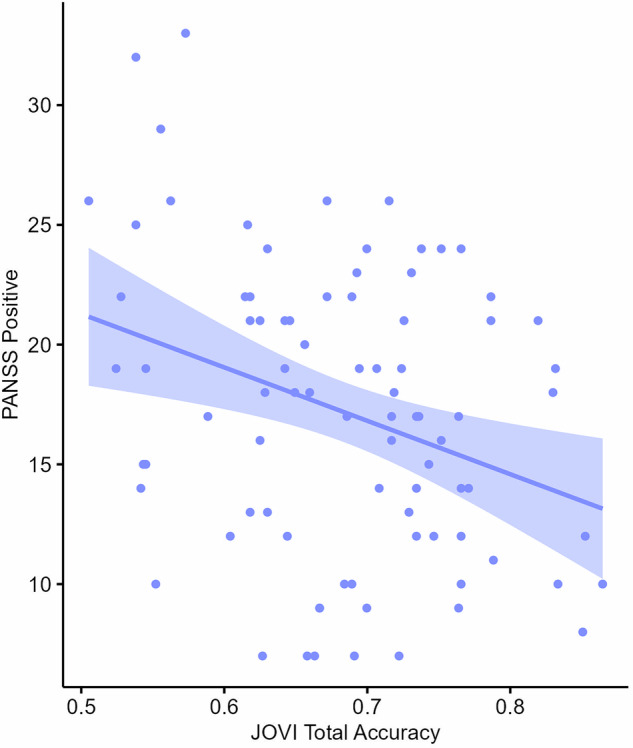
Correlation plot showing the association between total JOVI accuracy score and PANSS Positive subscale score for inpatients. Shaded band indicates 95% confidence interval.

Across the entire patient sample, combined Pearson’s correlation analyses with Benjamini-Hochberg correction revealed a statistically significant association between JOVI total percentage accuracy and PANSS Positive score (*r* = −0.32, *p* < 0.05; Fig. 4). There were no significant associations between JOVI total percentage accuracy and PANSS Total, Negative or General scores. This was also not significant for the more psychosis specific positive factor score from the 5-factor model^34^. Pearson’s and Spearman’s correlation analyses revealed no associations between JOVI threshold score and PANSS Total, Positive, Negative or General scores. Similarly, when we next examined schizophrenia spectrum group patients only, JOVI total accuracy was significantly negatively correlated with the positive factor score (*r* = −0.35, *p* < 0.05). Finally, Spearman’s correlation analyses between JOVI total percentage accuracy and individual PANSS items demonstrated no significant associations following Benjamini-Hochberg correction.

### IQ

For the inpatient groups, Spearman’s correlation analyses with Bonferroni corrections revealed a significant positive relationship between JOVI total percentage accuracy score and NART-estimated WAIS-IV IQ (*r* = 0.40, *p* < 0.001). There was also a significant positive relationship between JOVI threshold score and NART-estimated WAIS-IV IQ (*r* = 0.27, *p* < 0.01). A Spearman’s correlation analysis revealed no significant association between JOVI task performance and IQ in healthy controls.

### Task Specific Attention

Kruskal-Wallis analyses examining the effects of participant group on catch trial accuracy and non-responding found statistically significant between-group differences in both the target contiguous (χ^2^(2) = 15.32, *p* < .001, n^2^ = 0.11, 95% CI [0.04, 0.23]) and non-distractor (χ^2^(2) = 9.60, *p* < 0.01, n^2^ = 0.06, 95% CI [−0.00, 0.17]) catch trials only. Bonferroni corrected Wilcoxon rank sum tests revealed healthy controls had significantly higher accuracy in the target contiguous catch trial paradigm than both other psychiatric (*p* < 0.01, *r* = 0.31, 95% CI [0.13, 0.49]) and schizophrenia spectrum patient groups (*p* < 0.001, *r* = 0.43, 95% CI [0.24, 0.60]), as well as significantly higher accuracy in the non-distractor catch trial paradigm than the schizophrenia spectrum patient group (*p* < 0.01, *r* = 0.33, 95% CI [0.13, 0.52]).

### Digit span

**Fig. 5:**
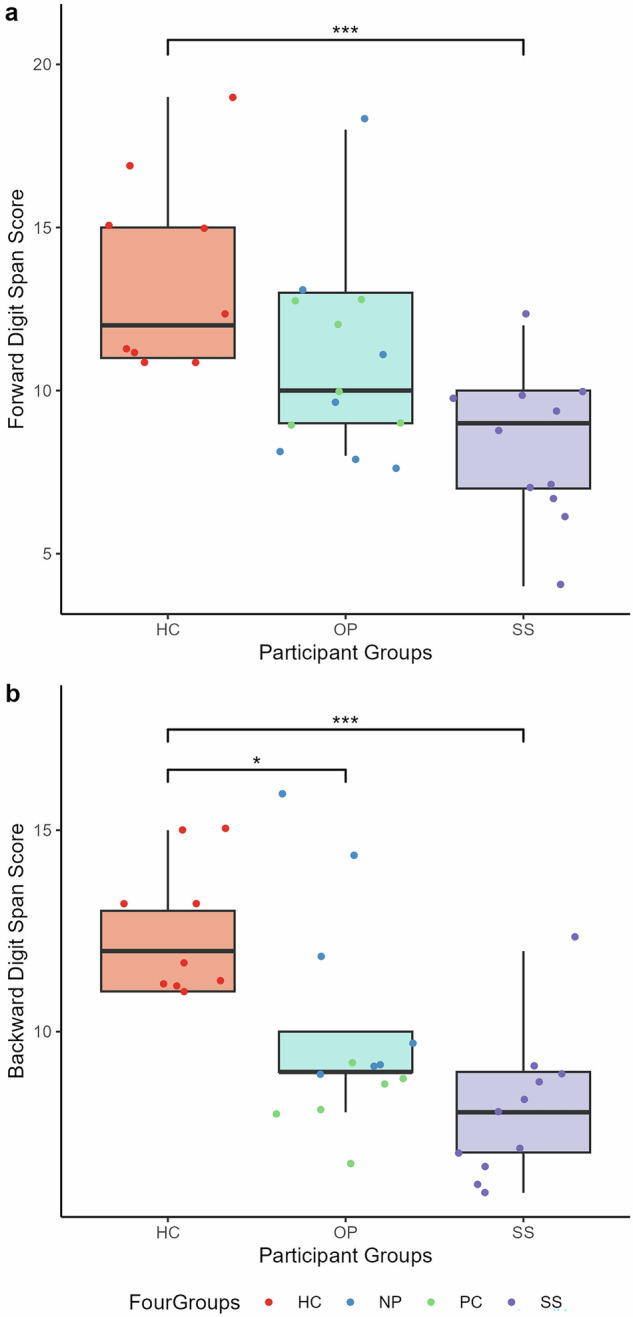
Group comparisons of Digit Span performance. **a** Digit Span Forward score and **b** Digit Span Backward score participant group comparisons. HC = healthy controls, OP = other psychiatric inpatients, SS = schizophrenia spectrum inpatients, NP = non-psychosis inpatients, PC = psychosis control inpatients. Note the dot colouring in the legend is for illustrative purposes only, and NP and PC groups were combined into the OP group for statistical analyses. Boxplot centre line represents median, lower hinge represents 25th percentile, upper hinge represents 75th percentile, lower whisker represents smallest value at most 1.5 × interquartile range (IQR) from the lower hinge, upper whisker represents largest value at most 1.5 × IQR from the upper hinge.

ANOVAs examining the effects of participant group on Digit Span Forward and Digit Span Backward scores found statistically significant between-group differences in both Forward (F(2, 30) = 9.24, *p* < 0.001, n^2^g = 0.38, 95% CI [0.14, 1.00]) and Backward (F(2, 30) = 11.19, *p* < 0.001, n^2^g = 0.43, 95% CI [0.19, 1.00]) paradigms (Fig. 5). Tukey post-hoc analyses showed healthy controls had higher Forward scores than schizophrenia spectrum inpatients (*p* < 0.001, 95% CI [−8.32, −2.25]), and higher Backward scores than schizophrenia spectrum (*p* < 0.001, 95% CI [−6.76, −2.13]) and other psychiatric (*p* = 0.024, 95% CI [−4.03, 0.19]) groups. These significances are lost when controlling for years of education, NART-estimated WAIS-IV IQ and chlorpromazine equivalents in both Forward and Backward paradigms. Pearson’s correlation analyses with Bonferroni corrected p-values revealed a statistically significant association between JOVI total accuracy and Digit Span Forward score (*r* = 0.62, *p* < 0.001) and Digit Span Backward score (*r* = 0.58, *p* < 0.01). Although the number of participants for which Digit Span data were collected was relatively low (see Table 1), the ANOVA findings are consistent with cognitive factors playing a role in the JOVI findings, and consistent with the observed correlation between JOVI and PANSS Positive scores.

### Structural equation modelling (SEM)

To further explore the relationship between JOVI performance and more generalised deficits associated with reduced IQ and Digit Span performance, SEM was conducted to assess the “bottom-up” impact of sensory integration (JOVI) on IQ and Digit Span as well as the reverse “top-down” impact of IQ and Digit Span on JOVI. Both the bottom-up model based on the previous literature (CFI = 1.00, TLI = 1.00, RMSEA = 0.00, 90%CI [0.00, 0.00], SRMR = 0.00, AIC = 771.42, BIC = 789.38, R2 = 0.53) and the opposite top-down model based on the broader visual science literature (CFI = 1.00, TLI = 1.00, RMSEA = 0.00, 90%CI [0.00, 0.00], SRMR = 0.00, AIC = 775.42, BIC = 796.37, R2 = 0.63) demonstrated acceptable goodness of fit indices, in the Digit Span Backward paradigm. A similar pattern of results was seen for Digit Span Forward (see Supplementary Results and Supplementary Fig. 1).

**Fig. 6:**
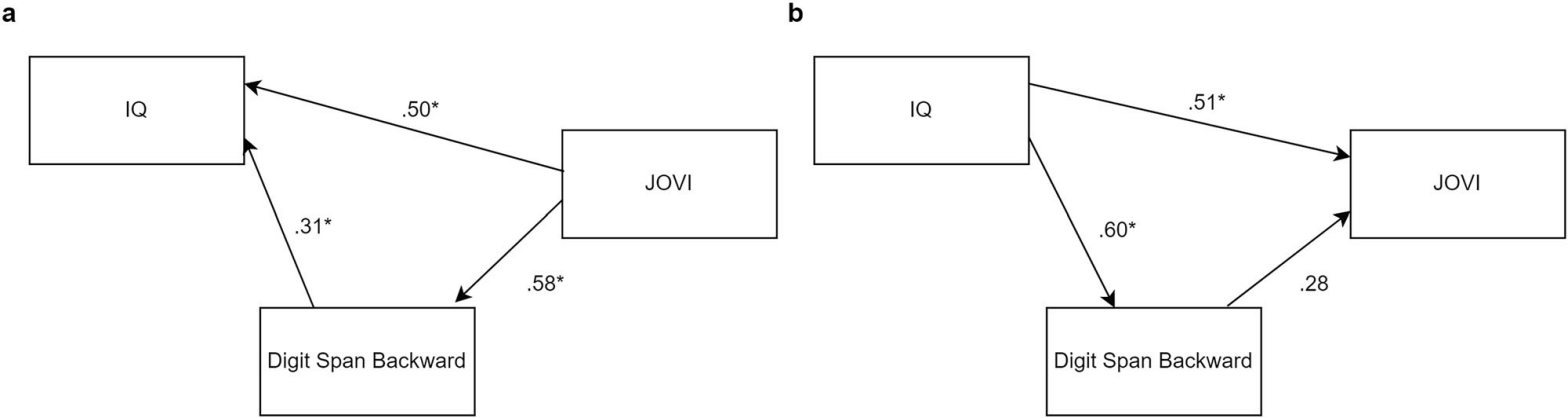
SEM of JOVI, IQ, and Digit Span Backward relationships. **a** Bottom-up and **b** top-down model, capturing the relationship between JOVI, IQ, and Digit Span Backward.

The bottom-up model demonstrated significant relationships between JOVI and Digit Span Backward (β = .58, *p* < 0.001), Digit Span Backward and IQ (β = 0.31, *p* < 0.05), and JOVI and IQ (β = 0.50, *p* < 0.01) (Fig. 6a). Additionally, the significant total effects of JOVI on IQ (β = 0.67, *p* < 0.001) and Digit Span Backward (β = 0.58, *p* < 0.001), and Digit Span Backward on IQ (β = 0.31, *p* < 0.05) support a mediation role of Digit Span Backward in the relationship between JOVI and IQ. The alternative top-down model also demonstrated significant relationships between IQ and Digit Span Backward (β = 0.60, *p* < 0.001), and IQ and JOVI (β = .51, *p* < 0.01) (Fig. 6b). Additionally, there were total effects of IQ on Digit Span Backward (β = 0.60, *p* < 0.001), and IQ on JOVI (β = 0.67, *p* < 0.001).

## Discussion

Visual integration deficits have been well-established in schizophrenia, though there is a paucity of research investigating the diagnostic specificity of the JOVI task to schizophrenia spectrum disorders. To address this gap, the present study assessed visual integration in schizophrenia spectrum, other psychiatric inpatients, and healthy controls to determine the relationship between visual integration and symptomatology.

A major finding of this study was that psychiatric groups showed impaired visual integration performance relative to controls. However, no difference was seen in performance between the schizophrenia spectrum and the other psychiatric diagnosis groups (in both overall JOVI accuracy and JOVI threshold score). These findings have been recently confirmed in an online sample assessed by the research team, similarly showing no significant distinction in visual integration performance between schizophrenia and other psychiatric disorders^32^. As visual integration performance is used as a phenotypic proxy for altered cortical neural integration, this finding not only raises questions about the diagnostic value of the JOVI task but also the neural factors that uniquely underpin schizophrenia spectrum disorders.

The current study also demonstrated a significant correlation between JOVI overall accuracy and PANSS Positive subscale scores, and the Positive factor from the 5 Factor model when only patients with psychosis were included, suggesting that psychosis independent of diagnosis is associated with total JOVI percentage accuracy. These findings may illustrate a possible association between visual integration and positive symptomatology. This aligns with previous work^35^ showing a similar association between visual motion integration impairment and positive symptoms, including the PANSS Delusions, Conceptual Disorganisation and Grandiosity items. Although significant correlations with specific symptoms were found between the Delusions, Conceptual Disorganisation, Excitement, Grandiosity, and Preoccupation items, these did not remain after post-hoc correction in the current study.

Taken together, the above findings align with the Research Domain Criteria (RDoC) approach that impairment is not specific to disorders. Consistent with the RDoC approach, the current study’s findings imply that visual integration impairment may not be specific to disorders and instead may be broadly linked to symptoms, like conceptual disorganisation. This highlights the need for broader approaches in future research, looking at cognitive-perceptual function across diagnoses and specifically the psychosis component, in order to truly comprehend the core factors involved.

The study further aimed to examine the JOVI task’s validity as a measure of visual-integration ability in the face of potential task confounds. Our results showed significant correlations between JOVI overall accuracy and threshold scores and NART-estimated WAIS-IV IQ, which, alongside the finding that healthy controls showed higher IQ than both inpatient groups, suggests that performance on the JOVI may be associated with general cognitive functioning. Although previous research by Keane et al. (2012)^27^ demonstrated lower IQ in schizophrenia and FEP participants compared with healthy controls, another study by Keane et al. ^36^ did not find IQ differences between schizophrenia participants and healthy controls. Additionally, measures of IQ did not correlate with contour integration performance in both aforementioned studies^27,36^, in contrast to the current study.

Our results also showed significant correlations between JOVI overall accuracy and threshold scores and Digit Span Forward and Digit Span Backward scores, indicating that reduced attentional and working memory ability were also associated with impaired JOVI performance. In addition, healthy controls showed significantly higher JOVI accuracy than the inpatient groups in both catch trial paradigms, and the other psychiatric inpatient group had significantly higher asymptotes than healthy controls, indicating an element of task-specific attentional difficulty. No significant between‐group catch-trial differences were found in two prior studies^37,38^, however the asymptote finding has been demonstrated in schizophrenia patients once previously^33^ and potentially reflects attentional lapses during the task. Additionally, our results showed that healthy controls had higher Digit Span Forward scores than schizophrenia spectrum inpatients and higher Digit Span Backward scores than schizophrenia spectrum and other psychiatric groups. To further assess the possible the possible influence of more general cognitive effects on JOVI SEM was conducted to assess both the bottom-up influence of visual integration (JOVI) on down-stream processes associated with working memory (Digit Span) and general intelligence (IQ) as well as the alternative top-down effects in the opposite direction. The SEM results demonstrate that both the bottom-up and top-down model performed comparably well with evidence of an influence in both directions.

The above findings demonstrate that there seems to be an element of attentional and working memory processing impacting participant performance, implying that visual integration performance may not be as immune from cognitive and attentional influences as previously believed when the CNTRACS initiative first developed the JOVI task^20^. While the SEM results provide a clue that there may be involvement of both top-down and bottom-up influences, the current sample lacks the statistical power needed to clarify if one direction is more influential. Together, these findings highlight the need for future investigations of tasks like JOVI to thoroughly explore attentional, working memory and other cognitive factors on performance. It may be oversimplistic to consider these influences as representing independent confounds in the data. As visual integration was selected because of the direct links to underlying neural integration in cortical networks, the fact that the JOVI task is designed to selectively engage visual cortical networks does not rule out the possibility that higher-level cortical networks are equivalently impacted by altered neural integration. The findings, highlight the difficulty in teasing apart bottom-up and top-down influences in task performance in clinical populations.

Potential limitations of the study include the heterogeneity of the inpatient participant group, and the use of medications and acute symptoms in the inpatient group. This extends to diagnostic ascertainment which was based on DSM-5 criteria but not using a standardised diagnostic tool. Additionally, specific psychiatric medications prescribed in the inpatient group are unknown, as only chlorpromazine and diazepam equivalent doses are known. However, no association was found between task performance and medication dose, likely mitigating the confounding effect of the medication. Future studies, sufficiently powered, could be undertaken to examine effects specific for depressive, manic or other symptom clusters. Also, future research with inpatient samples could incorporate following this group up as outpatients to determine if task performance varied with symptom resolution. Additionally, we are aware of the small sample size of the Digit Span data. Unfortunately, the Digit Span was included in the protocol after initial recruitment had already begun, and as a result only a subset of the participants had data for this task. Future research could expand upon the Digit Span findings with a larger sample.

Together this study provides fresh evidence of the overlapping complexities that exists across psychiatric diagnostic boundaries as well as the perception vs cognition boundaries that are often applied to distinguish function and cortical specificity. As with the increasing appreciation of the complex genetic interdependencies in psychiatric disorders^39,40^, the presumed selectivity of perceptual and cognitive deficits requires further interrogation. Understanding the associations between symptomatology, biology and perceptual and cognitive functioning in psychiatric disorders will be critical to future efforts to identify meaningful biomarkers and therapeutic targets.

## Methods

### Participant information

Ninety‐eight inpatients with a range of psychiatric disorders were recruited from the Monash Medical Centre (*n* = 32) and the Northern Hospital (*n* = 66) adult acute inpatient units in Melbourne, Australia. Thirteen participants were excluded for having achieved less than or equal to 50% total accuracy on the JOVI task^27,32^ (*n* = 6), lost data (*n* = 2), and incomplete PANSS or JOVI measures (*n* = 5) resulting in a final sample of *n* = 85. Forty-three healthy controls were recruited through community advertisement.

Psychiatric inpatients were split into two groups, schizophrenia spectrum (*n* = 40) and other psychiatric disorder (n = 45) without active psychosis, based on current diagnosis. A heterogeneous psychiatric comparator group without psychosis was chosen as other symptom domains were not of primary interest for this study. The schizophrenia spectrum disorder group was comprised of first episode psychosis (*n* = 2), psychotic disorder NOS (*n* = 1), schizoaffective disorder (*n* = 13), schizophrenia disorder (*n* = 24) patients, and the other psychiatric disorder group was comprised of bipolar affective disorder (BPAD; *n* = 18), BPAD with psychotic features (*n* = 1), BPAD with substance-induced psychotic disorder (SIPD; *n* = 1), borderline personality disorder (BPD; *n* = 1), brief psychotic disorder with peripartum onset (*n* = 1), major depressive disorder (MDD) with psychotic features and BPD (*n* = 1), SIPD (n = 1), substance use disorder (SUD) and SIPD (*n* = 1), SUD (*n* = 1), SIPD and BPD and MDD (*n* = 1), persistent depressive disorder (*n* = 1), MDD and PTSD (n = 2), MDD (*n* = 8), MDD with anxiety (*n* = 1), MDD and PTSD and BPD (*n* = 1), BPD and MDD (*n* = 1), alcohol use disorder (*n* = 2), and adjustment disorder (*n* = 2) patients. Diagnoses were retrieved from medical records and confirmed by treating psychiatrists in alignment with DSM‐V criteria^41^. Exclusion criteria comprised safety risk as assessed by the case clinician, intellectual disability defined as IQ < 70, acquired brain injury, history of neurological disorders, previous head surgery, receipt of electro‐convulsive therapy (ECT) within the six months preceding testing, and drug or alcohol dependence in the past 12 months. In addition to the above, healthy control participant exclusion criteria included previous psychiatric disorder diagnosis, previous suicidal ideation requiring treatment, current or previous use of antipsychotic medication, and use of psychotropic substances within the 14 days preceding testing. All participants included in the study had normal or corrected‐to‐normal vision, spoke English at a conversational level, and were aged between 18 and 65 years. Inpatient capacity to provide informed consent to research was assessed by both treating psychiatrists and researchers, and all participants provided written informed consent. Participants received financial compensation for their time.

The psychiatric inpatient component of the study was approved by the Monash Health (Project no.16099 A) and Melbourne Health Human Research and Ethics Committees (Project no. 2008.657), and the healthy control participant component was approved by University of Melbourne Human Research Ethics Committee (Project no. 1135478).

### JOVI task

Visual integration was assessed using the JOVI, which required participants to report the leftward- or rightward-pointing direction of a fragmented egg-shaped contour embedded in a Gabor element array. Participants indicated the direction in which the target was pointing by pressing either the left or right arrow key on a keyboard. Responses were recorded within the two seconds in which the stimulus was displayed, and otherwise coded as a missed response.

The stimulus comprised an 8 × 8‐cm grey square containing eighteen Gabor elements forming the non‐contiguous outline of the target stimulus and 298 randomly oriented distractor elements. The orientation alignment between the Gabors comprising the target was experimentally manipulated as jitter (Fig. 1). As the jitter level was increased, angular coherence between adjacent Gabors decreased; higher jitter levels placed increased demands on contour‐integration processing for target identification, and thus increased task difficulty. Jitter magnitude was incrementally increased from 0° (no jitter), to ±7°–8°, 9°–10°, 11°–12°, 13°–14°, and 15°–16°. Visual integration ability was assessed on the basis of participants’ accuracy-rate (i.e., proportion of correct responses) on jitter trials relative to alterations in the degree of orientation of target elements.

Participants completed a total of 288 trials in blocks of 12 trials each. Six blocks were repeated four times each, for a total of 24 trial blocks. Each trial block comprised a single jitter condition and subsequent blocks increased in difficulty. In each trial, a fixation screen was presented for one second prior to the stimulus. The fixation screen comprised a white fixation cross on a black background. Each block also contained a catch trial which, in alternating blocks, comprised either a continuous black target outline amongst distractor elements or a non‐jittered stimulus in the absence of distractors. These 48 catch trials were included to assess for participants’ inattention.

### Clinical measure

Inpatients were administered the PANSS to assess symptomatology across positive syndrome, negative syndrome, general psychopathology, and composite scales^42^ including the positive factor from the 5-factor model^34^. The PANSS is a well‐validated semi‐structured clinical interview for the assessment of both positive and negative symptoms in schizophrenia, as well as associated general psychopathology^43^.

### General intelligence

Participants completed the NART^44^, which was used to estimate a full‐scale intelligence quotient (FSIQ). NART error scores were then converted to standardised estimates of WAIS‐IV IQ for each participant^45^.

### Attention and working memory tasks

The Digit Span subtest from the WAIS‐IV was used to assess attentional and working memory ability, using the ‘forward’ task paradigm and ‘backward’ task paradigm, respectively. Participants were required to verbally repeat digit sequences of progressively increasing length, in the same order as administered for the Forward task paradigm, and in reverse order for the Backward paradigm. While the overall Digit Span score predominantly captures working memory ability (short‐term retention and mental manipulation of information), the comparatively simplistic Forward paradigm captures general basic attentional ability^46–48^. Raw scores were converted to scaled scores for subsequent analyses^49^.

### General procedure

The demographics questionnaire was first administered to all participants to screen for participant eligibility. This was followed by the NART and the Digit Span forward and backward tasks. Participants then commenced the JOVI task, after either achieving above 80% accuracy on a practice block of 10 trials with a 5°–6° jitter magnitude or completing five practice blocks. The JOVI was viewed by participants on an LCD laptop screen subtending approximately 43 × 27 degrees of the visual angle (1920 × 1080 pixels; 31.0 × 17.5 cm). The laptop was raised to ensure that the middle of the screen was aligned with participants’ eye level at a viewing distance of approximately 61 cm. As with previous applications of the task in clinical settings, no chin rest was used^28^. Participants were offered a 30 min break midway through the JOVI assessment. The PANSS was then administered to inpatients. To ensure reliability of PANSS ratings, cross‐referencing of symptom scoring was achieved by having two researchers present at each interview. Across recruitment drives, the overall study duration was approximately 2.5–3.0 h for inpatients and 1.0–1.5 h for healthy controls. Participants were assessed in similar testing environments. Additionally, participants were reimbursed between $15 and $20 for each hour of their time. The JOVI program was run in E-prime 2.0.

### Statistical analysis

Non-parametric Kruskal-Wallis and Chi-square tests were used to assess group differences in respective non-normally distributed continuous and categorical demographic variables. A two-way mixed ANOVA was performed to assess group differences in JOVI accuracy at different jitter trials, and a one-way ANOVA was employed to assess group differences in Digit Span subscales, followed by Tukey post-hoc analyses. Non-parametric Kruskal-Wallis tests were also used to assess group differences in jitter slope, jitter threshold, upper asymptote parameter, catch trial accuracy and non-responding, with Bonferroni post-hoc analyses. Independent *t*-tests were performed to determine inpatient group differences on PANSS subscale scores.

Pearson’s and Spearman’s correlation analyses were performed to determine relationships between JOVI performance and PANSS subscale scores, individual PANSS items, IQ, and Digit Span performances, with Bonferroni and Benjamini-Hochberg corrections.

Path analysis was conducted using SEM to evaluate both existing and proposed relationships between JOVI, IQ, and Digit Span Forward and Backward. Model fit was assessed through several goodness-of-fit indices: the Root Mean Square Error of Approximation (RMSEA; acceptable value < 0.08), the Standardized Root Mean Square Residual (SRMR; acceptable value < 0.1), the Comparative Fit Index (CFI; acceptable value > 0.9), the Tucker-Lewis Index (TLI; acceptable value > 0.9), along with the Akaike Information Criterion (AIC), Bayesian Information Criterion (BIC), and R² values.

Analyses were two-tailed with α = 0.05. No extreme outliers were identified in the data. The data analysis was performed using the software package R version 4.2.1. The SEM was performed using Stata software version 18.0.

## Supplementary information

Supplementary Figure 1

Supplementary Results

## Data Availability

The datasets used and analysed during the current study are available from the corresponding author on reasonable request.
