# Supplementary material for: Visual integration deficits associated with psychosis are independent of diagnosis
Source: Schizophrenia (Heidelb). 2025 Apr 9;11(1):58. doi: 10.1038/s41537-025-00606-0 (PMC11982286; doi:10.1038/s41537-025-00606-0)
Supplement: Supplementary file 1 — Supplementary Figure 1 [file 41537_2025_606_MOESM1_ESM.docx]

**Supplementary Figure**


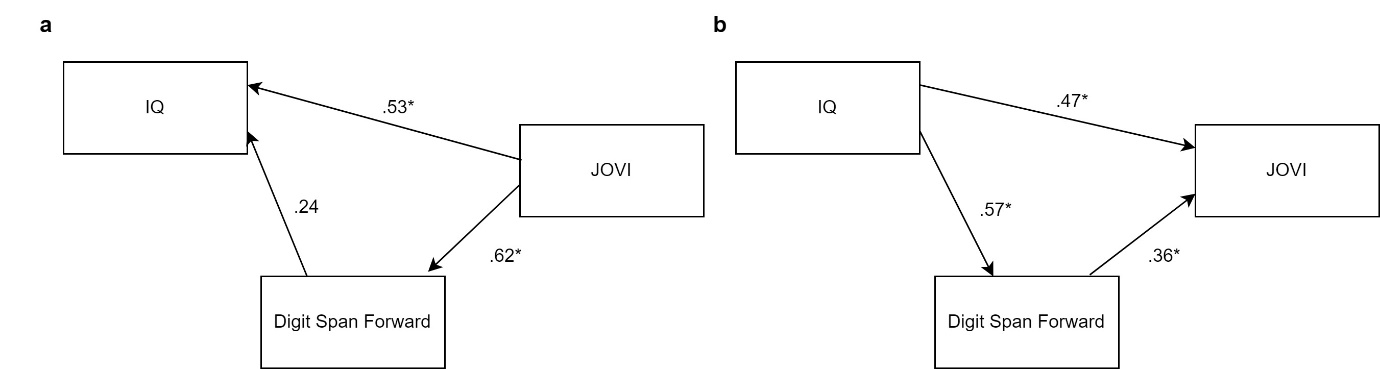


**Supplementary Figure 1.** Structural equation modelling using the Digit Span Forward paradigm.
(**a**) Bottom-up model and (**b**) top-down model, capturing the relationship between JOVI, IQ, and Digit Span Forward.
