# Supplementary material for: Visual integration deficits associated with psychosis are independent of diagnosis
Source: Schizophrenia (Heidelb). 2025 Apr 9;11(1):58. doi: 10.1038/s41537-025-00606-0 (PMC11982286; doi:10.1038/s41537-025-00606-0)
Supplement: Supplementary file 2 — Supplementary Results [file 41537_2025_606_MOESM2_ESM.docx]

**Supplementary Results**

**Structural Equation Modelling: Digit Span Forward**

Both the bottom-up model, based on previous JOVI literature, (CFI = 1.00, TLI = 1.00, RMSEA = 0.00, 90%CI [0.00, 0.00], SRMR = 0.00, AIC = 786.47, BIC = 804.43, R^2^ = 0.55) and the top-down model, based on the current paper and broader visual task literature, (CFI = 1.00, TLI = 1.00, RMSEA = 0.00, 90%CI [0.00, 0.00], SRMR = 0.00, AIC = 790.47, BIC = 811.42, R^2^ = 0.60) demonstrated acceptable goodness of fit indices, in the Digit Span Forward paradigm. The top-down model demonstrated slightly better model predictions.

The bottom-up model demonstrated significant relationships between JOVI and Digit Span Forward (β = .62, *p* < .001), and JOVI and IQ (β = .53, *p* < .01). Additionally, there were significant total effects of JOVI on IQ (β = .67, *p* < .001) and JOVI on Digit Span Forward (β = .62, *p* < .001). The top-down model demonstrated significant relationships between IQ and Digit Span Forward (β = .57, *p* < .001), Digit Span Forward and JOVI (β = .36, *p* < .05), and IQ and JOVI (β = .47, *p* < .01). Additionally, there were significant total effects of IQ on JOVI (β = .67, *p* < .001), IQ on Digit Span Forward (β = .57, *p* < .001), and JOVI on Digit Span Forward (β = .36, *p* < .05).
